# Medical Supervision of the Coal Tar Industries

**Published:** 1918-10

**Authors:** F. G. Möhlau

**Affiliations:** Buffalo, N. Y.


### BUFFALO MEDICAL JOURNAL

Yearly Volume 74 OCTOBER, 1918 Number 3

### ORIGINAL ARTICLES

The right is reserved to decline papers not dealing with practical medical and surgical subjects, and such as might offend or fail to interest readers. Contributors are solely responsible for opinions, methods of expression and revision of proof.

### Medical Supervision of the Coal Tar Industries.

By DR. F. G. MÖHLAU, Buffalo, N. Y.

Protection of health of the working men engaged in the manufacture of Coal Tar Products is the most important problem facing the management of the Chemical Industries. The health of the worker naturally is a great asset to the successful maintenance of equilibrium.

It must be remembered that the Coal Tar industry is, comparatively speaking, a new one in this country, and the welfare of the worker must be scrupulously guarded along the most approved lines compatible with factory administration. The greatest care must be taken in introducing reforms and changes to obtain maximum results and reduce expense.

Health is the primary factor for the efficient performance of the working man's duties. Sickness decreases the worker's efficiency and production, and increases the risk of accidents in the various departments. An epidemic can disorganize a department, even the whole force of the factory. We have to consider outside influences, (environment, fatigue, accident), and inside influences (disease and defects).

Surroundings in factory work, such as light, ventilation and dust, influence the efficiency of the workers to a great extent, and to this may be added humidity and heat, also cold and drought.

In all human, or, broadly speaking, muscular activity, carbon dioxid is produced. With hard work, double the amount is produced and thrown off with the expired air. Toxic proteins are also thrown off of the working body. These toxic products are the odors we sense in an ill-ventilated (close) room. The headache, tired feelings, lassitude and malaise experienced in close surroundings, we must attribute to heat and humidity rather than to the inspiring of waste products. The effect of heat and moisture on the skin we must consider as the determining cause of discomfort. From these facts we gather that the working quarters must have a free circulation of fresh air, also that the humidity of the air surrounding the worker must be within certain limits in working quarters. It should have not more than six per 10,000 parts of CO2, the temperature should not rise above 68° F, and the humidity should not be higher than 72 wet bulb.

Good light is not only a prima facie factor in the health of the workman, but also in the efficiency of his work. General semi-direct illumination supplemented where necessary by individual shaded lights probably gives best results. Each worker, or group of workers, can thus regulate the light supply. The light should fall on the work and not on the workers' eyes. Thus, three dangers would and should be avoided: (1) over-illumination, which is liable to cause irritation of the retina or contracted iris, (2) under-illumination, causing eye strain, (3) reflected light on bright surface or colors tends to confuse and produce effects of over-illumination. Poor illumination, besides injuring the worker, reduces output and increases the number of accidents.

Fatigue should be carefully guarded against as it decreases capacity for work, and follows excess of work or lack of rest. No subject that has to do with industrial health has received more thought and attention than industrial fatigue. Especially since the beginning of the war the question of fatigue has found serious consideration by the ministers of war munitions all over Europe. From a physiological standpoint, we have for instance the poisons produced by the contraction of the muscular fibres, especially the voluntary muscles, the changes of glycogen in the muscles, and the consumption of the stored-up glycogen in the muscles and liver. Even physiologically these substances are of an acid reaction due to carbon dioxid and sarcolactic acid. These are the factors producing fatigue, not only causing a tired feeling in the muscle, but also in the nerves and nerve centers. These waste products are eliminated slowly or gradually during the period of rest which, naturally, is necessary for the repair of body waste, and for this reason it must be understood that rest during the working days is necessary. If sufficient rest is not allowed a deficit is the result and we must consider this an injury to the working power of the following days. The strain of the work done after such symptoms are manifest, not only requires more effort, but also results in defective production. The effort increases, but still you must acknowledge that during such conditions the working man accomplishes less. Over-work must be guarded against. You must also consider that over-work, where fatigue is manifest, increases the danger of accident, and is the starting point of impairment of health.

As the type of work varies in different departments, the physical condition of each man is varying, and so will the efforts put forth vary and increase or diminish the working power.

Considering accidents, we always claim that they are largely due to carelessness on the part of the working man. But, while a great percentage of accidents are caused by carelessness, we must admit that in coal tar industry they are due to the complexity of the work rather as compared with the worker's ability and knowledge. The continuation of the work of any man we must consider as defective until he has completely recovered of such an accident. The proportion of accidents due to sickness or physical defects is generally considered rather low as records here will show. All accidents, no matter how minute, should therefore be treated by able assistance; injuries in any way serious should be attended by a trained surgeon. By prompt and efficient treatment is meant that the workman should be taken care of within at least thirty minutes of such injury.

Physical defects are an aggravating factor against health and a cause of accidents if the worker is not properly placed. A defective worker is not by any means an inefficient worker, and inefficiency only occurs when the physical defect interfers with the power of producing a normal output, especially by fatigue and discomfort. Defective workers are very often the best workers in certain departments, but they will not remain so unless properly supervised.

Sickness is prevalent among a large percentage of workers, is most marked during changeable weather or high temperature, and is an important factor in the loss of time. We can safely state that 2% of all working men at one time or another are absent on account of sickness. These conditions should most seriously be guarded against and all efforts should be made for the protection of the health of the working man. Absentees from work naturally decrease the output.

Considering the foregoing factors which control the health of the worker, it is evident that the responsibility for the workers' health falls on four men: the engineer of the plant, the safety engineer, the manager of the works, and the factory surgeon.

1. The engineer of the plant controls the external factors of environment.

2. The safety engineer controls the external factors of danger.

3. The manager of the works controls the external factors of fatigue.

4. The factory surgeon controls the internal factors of disease and physical defects.

No further consideration will be given the first three. Each is a specialty in itself.

We will now consider the work of the factory surgeon and the medical supervision of the workers.

The object of medical supervision is to control the health of the working force by instituting and maintaining methods of prevention of disease; by prompt attention to accidents and, through careful after-care, prevention of disability as far as possible; by finding defects in workmen and repairing them and if this is not possible, endeavoring to place the defective workman where he will be most efficient; by the education of the workman in matters of hygiene; and by the control of sanitation and matters pertaining to the health of the factory at large.

The essential knowledge of the physical condition of a working man is gained by the physical examination. Many a defect is discovered during the examination, and if the employee is placed in the proper department and the defect guarded against with efficiency the condition will gradually be improved. Workers can be enthused to co-operate with the physician or surgeon in helping those under supervision and enforcing his advice. The value of the physical examination to the factory and to the worker becomes plainer every moment.

Defectives may be divided into two classes: (1) those who can be easily relieved by medical supervision and placed accordingly, and (2) those who can be assisted by medical advice only.

The first class of defectives are those with defective eyes, ears, heart, lungs, and certain defects of the extremities.

Eyes and ears are of particular importance in office work. Slight refractive errors, if not corrected, lead to eye strain and poor work which can be of no advantage to anybody. Slight deafness among stenographers is liable to produce considerable nerve strain and such work should be carefully avoided. Defective hearts are factors of different moment. The average patient with a compensated heart is able to do considerable work without any trouble, yet a long strain should be avoided and all exercise should stop when troubles manifest themselves, to avoid exhaustion. Defective extremities are a less important factor and are not to be considered in this short resume.

Of course, it must naturally be seen to that defectives are placed in suitable departments. Medical supervision, therefore, should actually begin with the primary examination, and each worker known to be defective should be reexamined at reasonable intervals as long as he is working at the same factory, and careful advice given him during the period of disability and strict instructions during his working time. Medical supervision should preserve the general health of the working man and should be a most prominent factor in educating the worker how to live, and opportunities should be given for interviews between such defective workers and the factory physician or surgeon.

The great bulk of sickness among working men is of minor character, yet unless carefully treated may lead to more serious conditions, which naturally should be avoided by all possible means. If we consider the time lost in working hours, this becomes very apparent.

Not only this, but also the welfare of the working man's whole family should be at heart of the employer, and great benefits may be derived by giving him an opportunity to consult with the factory physician as to the health of himself and family; for even his family troubles will influence the efficiency of the working man. By giving a helping hand, faith, satisfaction and an effort to do better work will be the results.

In transferring a man from one department to another, which should be out of the question without a physical examination, the problem is to allot work in harmony with the man's constitution. While ailments of the worker may be of small importance in one branch of the factory, the health or welfare of the man may be jeopardized by changing about to another department.

One more serious and important factor in the welfare of a factory is the care and consideration the worker receives from his Superior and from the Medical Department, to which the welfare department must be subservient if results are to be obtained.

The best index as to sickness, number of accidents, and the character thereof, is the record of lost time. Sickness and accidents among workers are almost equally prevalent and should receive the same care and attention. I consider that when the time lost for personal reasons and time lost for sickness are about even, or the time lost for personal reasons somewhat greater, good medical service is being provided. So far, I am sorry to say that no such records are being kept, but I am sure that the time lost without given reason is enormous. Deficient medical service and inadequate welfare are the great factors in the permanent number of floating workers. Good results can be obtained only by making the men feel protected and keeping a careful check on each case of sickness and accident, and by an efficient follow-up system keeping in close personal touch. It is the keynote of success to keep the heart and confidence of the worker.

The whole question of the preservation of the health of the worker in hazardous surroundings evolves itself into education and service:—education of the employer as to the economy of preserving the health of his employee and education of the employee as to how to live and how to avail himself of proper medical supervision. Once this education is accomplished, success is assured, and the migrating worker eliminated. The result will be efficient service and a faithful employee.

The instruments of service are the Industrial Physician, Nurse and Hospital. The Industrial Physician now must be considered a specialist, and as it is well understood, one of high order and value, whose development has taken time and patient study. His equipment and training are special (should be), and more closely approximate those of the Military Surgeon than any other branch of the profession. His responsibilities have been outlined to some extent in these paragraphs and it must be evident from what has here been put before you that his responsibilities are great, and beyond dispute. Personally, I have put forward an effort to solve the problems and demands of the medical needs in the Chemical Industry that has been altogether new in this country, and in order to do justice to the service much improvement is necessary.

Natl. Aniline & Chem. Co., Buffalo, N. Y.

---

**Bullet Penetrating Aorta and Lodging in Femoral Artery.** Roualt, Le Progres Med., Jan. 26, describes the case of a soldier wounded in the right scapular region. Motor and sensory paralysis of the leg, haemothora and haemoptysis, beginning gangrene of the leg were the principal manifestations. The ball was at first missed by radiography but later found in the triangle of Scarpt and was located within the femoral artery and removed. Death, however, followed and, on necropsy, it was found that it had splintered the fourth rib, traversed to lower lobe of the lung, entered the orata just below the arch and had lacerated the internal coat on the opposite side, the wound of entrance into the aorta having healed. The ball then advanced until the gradually diminishing calibre of the arterial branches stopped it, with shutting off of the circulation, the sudden ischaemia of the nerves rather than any actual traumatic lesion, determining the paralysis.

